# Evaluation of p16/Ki-67 dual staining in detection of cervical precancer and cancers: a multicenter study in China

**DOI:** 10.18632/oncotarget.8307

**Published:** 2016-03-23

**Authors:** Lu-Lu Yu, Wen Chen, Xiao-Qin Lei, Yu Qin, Ze-Ni Wu, Qin-Jing Pan, Xun Zhang, Bai-Feng Chang, Shao-Kai Zhang, Hui-Qin Guo, You-Lin Qiao

**Affiliations:** ^1^ Department of Cancer Epidemiology, Cancer Hospital, Chinese Academy of Medical Sciences and Peking Union Medical College, Beijing, P. R. China; ^2^ Department of Pathology, Cancer Hospital, Chinese Academy of Medical Sciences and Peking Union Medical College, Beijing, P. R. China; ^3^ Department of Cancer Epidemiology, Henan Cancer Hospital, Affiliated Cancer Hospital of Zhengzhou University, Henan Office for Cancer Control and Research, Zhengzhou, P. R. China

**Keywords:** cervical cancer, p16/Ki-67 dual staining, human papillomavirus, Chinese women, Pathology Section

## Abstract

**Purpose:**

To analyze the clinical performance of p16/Ki-67 dual-stained cytology identifying high-grade cervical intraepithelial neoplasia (CIN2+) in Chinese women.

**Methods:**

1079 women attending ongoing cervical cancer screening and 211 “enriched” women aged ≥30yrs with biopsy-confirmed CIN2+ from five Chinese hospitals were enrolled during year 2014-2015. Cervical specimens were collected for high-risk human papillomavirus (HR-HPV) DNA analysis, Liquid-based cytology (LBC) and p16/Ki-67 dual staining. Colposcopy and biopsy were performed on women with any abnormal result.

**Results:**

p16/Ki-67 positivity increased with histologic severity. It was 18.4%(183/996) in normal histology, 54.0%(34/63) in CIN1, 81.0%(34/42) in CIN2, 93.3%(111/119) in CIN3, 71.4% (5/7) in adenocarcinoma and 95.2%(60/63) in squamous cell carcinoma. Compared with the HR-HPV negatives, p16/Ki-67 expression was significantly higher in the HPV16/18 positive (OR: 35.45(95%CI: 23.35-53.84)) and other 12 HR-HPV types positive group (OR: 8.01(95%CI: 5.81-11.05). The sensitivity and specificity of p16/Ki-67 to detect CIN2+ in the entire population were 90.9% and 79.5%, respectively. In women with ASC-US and LSIL, sensitivity and specificity for detection of CIN2+ were 87.5% and 66.4%, respectively, with a referral rate of 43.8%. In women who tested positive for HR-HPV, sensitivity and specificity of dual-staining for detection of CIN2+ were 92.7% and 52.7%, respectively, and the referral rate was 68.7%.

**Conclusions:**

p16/Ki-67 dual-stained cytology provided a high sensitivity and moderate specificity to detect underlying cervical precancer and cancers in various settings, and might be considered as an efficient screening tool in China.

## INTRODUCTION

As the most populous country in the world, the disease burden of cervical cancer varies greatly in China due to the unbalanced development of economy, uneven distribution of its population and screening coverage. More than 70% of the Chinese population living in rural areas where 90% of incident cervical cancer estimate to occur, however, cervical cancer screening is mainly available in cities, rural Chinese women receive minimal benefit from screening improvement [1]. From 2009 to 2015, the Chinese government launched a nationwide free cervical cancer screening project for 40 million rural women by using visual inspection with acetic acid (VIA) and cytology based screening methods, however, with an estimation of 500 million women in rural areas, cervical cancer remains a critical problem threatening women's health.

Papanicolaou (Pap) cytology is widely used for cervical cancer screening since its introduction to China in 1999. However, the positive prediction of atypical squamous cells of undetermined significance (ASC-US) and low-grade intraepithelial lesions (LSIL) cytology results for the presence of high-grade cervical intraepithelial neoplasia (HG CIN) is relatively low, with a 5-year risk for cervical intraepithelial neoplasia grade 3 or worse (CIN3+) of 2.6% and 5.2%, respectively [2]. So a method to identify women who harboring HG CIN with ASCUS/LSIL cytology is highly needed.

In resource limited rural areas in China, it is difficult to build the infrastructure necessary for a successful Pap cytology screening system. Alternative screening strategies have been developed, including molecular testing for HR-HPV -the necessary cause of cervical cancer. HR-HPV DNA testing has been evaluated and shown to be more sensitive and reliable than Pap cytology [3-7]. In April 2014, the FDA approved the use of an HPV test (the cobas HPV Test) for primary cervical cancer screening for women aged 25 years and older. However, though a negative HPV result can almost exclude that women have precancer or cancer [8, 9], 80%-90% of women who tested positive will not have concurrent diseases. Limited resources need to be prioritized for women at the highest risk for harboring cancer, the management of screen-positive women is a coming issue.

p16^INK4a^ (p16) is a cyclin-dependent kinase inhibitor that has been proven to be strongly overexpressed in transforming infections with oncogenic types of HPV and is believed to be a surrogate marker for precancerous cervical lesions [10, 11]. However, overexpression of p16 may not only be observed in dysplastic cells but also in tubal metaplasia and endometrial cells as well as in normal columnar cells from the cervix, which raises challenge in examining cytological samples. Ki-67 is a nuclear antigen and a cellular proliferation marker expressed in all cell-cycle phases except G0, which is also overexpressed in HG CIN [12]. In normal cells, the expression of p16 and Ki-67 is mutually exclusive. Hence, it is thought that the simultaneous detection of p16 overexpression and Ki-67 within a cell would be indicative of deregulation of the cell cycle and a transforming HPV infection which may progress to cancer. The CINtec PLUS Cytology test (Roche Tissue Diagnostics/Ventana Medical Systems, Inc., Tucson, AZ, USA) is an immunocytochemical cocktail composed of antibodies against p16 and Ki-67. Many studies have been conducted to assess the clinical utility of p16/Ki-67 dual-staining for the detection of HG CIN in primary cervical screening or in ASCUS/LISL triage as well as for the triage of Pap cytology-negative, HPV positive screening results [13-15], but no such data were available in China. We performed this study to evaluate the clinical utility of p16/Ki67 as a marker for detecting cervical precancer and cancer in a combined population of women referred from colposcopy clinics and from a screening program.

## RESULTS

A total of 1,357 Chinese women were enrolled. Of them, 35 (2.6%) women were excluded due to incomplete histology results, 32 (2.4%) women were excluded because of indeterminate cobas HPV Test results or unsatisfactory cytology results. A total of 1,290 women were included in the final analysis, including 1,079 (83.6%) women from the screening group. Among them,481(44.6%) had abnormal test results and received colposcopy, with 63 CIN1, 14 CIN2 and 6 CIN3 cases. There were 211 women in enriched group, i.e. 16.4% of all women. The mean age of the women included in the analysis was 48.3 ± 8.7 years (range, 30-69 yrs; median: 48 yrs).

As shown in Table 1, the overall test positivity of p16/Ki-67 dual staining (33.1%) was a little lower than that of the HR-HPV (35.9%) and LBC (36.3%) in the whole population (*p* < 0.05). It increased significantly with disease severity (*p_trend_* < 0.0001). There were 996 (77.2%) women with negative histology, 63 (4.9%) with CIN1, 42 (3.3%) with CIN2, 119 (9.2%) with CIN3, 63 (4.9%) with SCC, and 7 (0.5%) with ADC. The corresponding p16/Ki-67 dual staining positivity was 18.4%, 54.0%, 81.0%, 93.3%, 95.2%, and 71.4%, respectively.

**Table 1:** p16/Ki67, HR-HPV and LBC positivity in cytology and histology categories

Category	N	p16/Ki67	HR-HPV	LBC(≥ASCUS)
		N(%)	N(%)	N(%)
All				
Total population	1290	427(33.1)	463(35.9)	468(36.3)
Screening population	1079	232(21.5)	265(24.6)	265(24.6)
Cytology				
Normal	822	130(15.8)	143(17.4)	-
ASCUS	179	58(32.4)	72(40.2)	-
ASC-H	35	19(54.3)	22(62.9)	-
AGC	8	5(62.5)	3(37.5)	-
LSIL	77	54(70.1)	64(83.1)	-
HSIL	109	103(94.5)	102(93.6)	-
SCC	56	54(96.4)	53(94.6)	-
ADC	4	4(100.0)	4(100.0)	-
Histology				
Normal	996	183(18.4)	204(20.5)	214(21.5)
CIN1	63	34(54.0)	41(65.1)	38(60.3)
CIN2	42	34(81.0)	40(95.2)	32(76.2)
CIN3	119	111(93.3)	111(93.3)	116(97.5)
SCC	63	60(95.2)	62(98.4)	62(98.4)
ADC	7	5(71.4)	5(71.4)	6(85.7)
CIN2+ (total population)	231	210(90.9)	218(94.4)	216(93.5)
CIN2+ (screening population)	20	15(75.0)	20(100.0)	13(65.0)
CIN2+ (enriched population)	211	195(92.4)	198(93.8)	203(96.2)
CIN3+ (total population)	189	176(93.1)	178(94.2)	184(97.4)
CIN3+ (screening population)	6	5(83.3)	6(100.0)	5(83.3)
CIN3+ (enriched population)	183	171(93.4)	172(94.0)	179(94.8)

^a^Abbreviations: ASCUS, atypical squamous cells- undetermined significance; ASC-H, atypical squamous cells cannot exclude high-grade squamous intraepithelial lesion; AGC, atypical glandular cells; LSIL, low-grade squamous intraepithelial lesion; HSIL, high-grade squamous intraepithelial lesion; SCC, squamous cell carcinoma; ADC, adenocarcinoma; LBC, liquid-based cytology; CIN, cervical intraepithelial neoplasia; CIN2+, cervical intraepithelial neoplasia grade 2 or worse; CIN3+, cervical intraepithelial neoplasia grade 3 or worse.

To analyze the associations between HR-HPV infection and p16/Ki-67 dual-stain positivity, the participants were divided into three groups according to HPV status. HPV positivity was strongly associated with p16/Ki-67 dual staining in the whole population (Table 2). Compared to HR-HPV negatives, p16/Ki-67 expression in HPV16/18 positive and other 12 HR-HPV types positive group was significantly higher, with an odds ratio (OR) of 35.45 (95% CI: (23.35-53.84)) and of 8.01 (95%CI:(5.81-11.05)), respectively. Specifically, when stratified by histology, the association was still significant (all *p* < 0.05).

**Table 2:** Association of p16/Ki67 expression and HR-HPV in different histology categories

		p16/Ki67 positive*N*(%)	p16/Ki67 negative*N*(%)	*P*Value	OR (95%CI)
All					
	HR-HPV negative (*n* = 827)	109(13.2)	718(86.8)	-	Ref
	Other 12 HR-HPV positive (*n* = 246)	135(54.9)	111(45.1)	<0.001	8.01(5.81-11.05)
	HPV16/18 positive (*n* = 217)	183(84.3)	34(15.7)	<0.001	35.45(23.35-53.84)
< CIN2					
	HR-HPV negative (*n* = 814)	101(12.4)	713(87.6)	-	Ref
	Other 12 HR-HPV positive (*n* = 183)	79(43.2)	104(56.8)	<0.001	5.36 (3.74-7.68)
	HPV16/18 positive (*n* = 62)	37(59.7)	25(40.3)	<0.001	10.45(6.04-18.08)
CIN2+					
	HR-HPV negative (*n* = 13)	8(61.5)	5(38.5)	-	
	Other 12 HR-HPV positive (*n* = 63)	56(88.9)	7(11.1)	0.021	5.00(1.28-19.60)
	HPV16/18 positive (*n* = 155)	146(94.2)	9(5.8)	0.001	10.14(2.75-37.37)

^a^Abbreviations: OR, Odds ratio; CI, confidence interval; CIN2+, cervical intraepithelial neoplasia grade 2 or worse.

_b_other 12 HR-HPV positive: positive for any of the 12 HPV types(HPV 31, 33, 35, 39, 45, 51, 52, 56, 58, 59, 66 and 68), and negative for HPV16/18.

Sensitivities, specificities, PPVs, NPVs, AUCs, and colposcopy referral rates for all screening methods to detect CIN2+ or CIN3+ are shown in Tables 3, 4 and 5. In the whole population, the sensitivity of p16/Ki-67 to detect CIN2+ and CIN3+ were 90.9% and 93.1%, respectively, which were similar with that of HR-HPV and LBC (all *p* > 0.05); the specificity of p16/Ki-67 to detect CIN2+ was similar with that of HR-HPV(*p* > 0.05), but slightly higher than that of LBC (79.5% *vs* 76.2%, *p* = 0.042);in detection of CIN3+, the specificity of p16/Ki-67 was not significant different with that of LBC (*p* > 0.05), but a little higher than that of HR-HPV (77.2% *vs* 74.1%, *p* = 0.032). The similar situation was also observed in the screening population.

**Table 3:** Clinical performance characters of p16/Ki67 dual staining, HR-HPV test and LBC for detection of CIN2+ or CIN3+ in entire population

	Sensitivity	Specificity	PPV	NPV	AUC
	%(95%CI)	%(95%CI)	%(95%CI)	%(95%CI)	(95%CI)
CIN2+					
Total population(CIN2+=231)					
p16/Ki67	90.9(86.5-94.0)	79.5(77.0-81.8)	49.2(44.5-53.9)	97.6(96.3-98.4)	0.852(0.826-0.878)
HR-HPV	94.4(90.6-96.7)	76.9(74.2-79.3)	47.1(42.6-51.6)	98.4(97.3-99.1)	0.856(0.832-0.880)
LBC	93.5(89.6-96.0)	76.2(73.6-78.7)	46.2(41.7-50.7)	98.2(97.0-98.9)	0.849(0.824-0.873)
Screening population(CIN2+ =20)					
p16/Ki67	75.0(53.1-88.8)	79.5(77.0-81.8)	6.5(4.0-10.4)	99.4(98.6-99.8)	0.773(0.662-0.883)
HR-HPV	100.0(83.9-100.0)	76.9(74.2-79.3)	7.5(4.9-11.4)	100.0(99.5-100.0)	0.884(0.851-0.917)
LBC	65.0(43.3-81.9)	76.2(73.6-78.7)	4.9(2.9- 8.2)	99.1(98.2-99.6)	0.706(0.584- 0.829)
CIN3+					
Total population(CIN3+ =183)					
p16/Ki67	93.1(88.6-96.0)	77.2(74.6-79.6)	41.2(36.7-45.9)	98.5(97.4-99.1)	0.852(0.825-0.878)
HR-HPV	94.2(89.9-96.7)	74.1(71.5-76.6)	38.4(34.1-43.0)	98.7(97.6-99.3)	0.841(0.815-0.867)
LBC	97.4(94.0-98.9)	74.2(71.5-76.7)	39.3(35.0-43.8)	99.4(98.6-99.7)	0.858(0.835-0.880)
Screening population(CIN3+ =6)					
p16/Ki67	83.3(43.7-97.0)	78.8(76.3-81.2)	2.2(1.0-4.9)	99.9(99.3-99.9)	0.811(0.638-0.984)
HR-HPV	100.0(61.0-100.0)	75.9(73.2-78.3)	2.3(1.0-4.9)	100.0(99.5-100.0)	O.879(0.821-0.937)
LBC	83.3(43.7-97.0)	75.8(73.1-78.2)	1.9(0.8-4.3)	99.9(99.3-99.9)	0.796(0.622-0.969)

^a^Abbreviations: CIN2+, cervical intraepithelial neoplasia grade 2 or worse; CIN3+, cervical intraepithelial neoplasia grade 3 or worse; LBC, liquid-based cytology; CI, confidence interval; PPV, positive predictive value; NPV, negative predictive value; AUC, area under ROC curve.

There were 256 women diagnosed as ASC-US or LISL in the whole population, and 48 CIN2+ cases were detected in this group; the p16/Ki-67 positivity was 43.8%, indicating that colposcopy referral would be reduced by more than half if p16/Ki-67 dual-stain were used as a triage test (Table 4). The sensitivity and specificity for detection of CIN2+ were 87.5% and 66.4%, respectively. The sensitivity and specificity for detection of CIN3+ were 89.7% and 62.1%, respectively. Compared to HR-HPV test, p16/Ki-67 dual staining had a similar sensitivity (*p* = 0.727 and 1.000) for both endpoints, but a higher specificity (*p* = 0.003 and 0.002).

**Table 4:** Clinical performance characteristics of p16/Ki67 dual staining and HR-HPV test for detection of CIN2+ or CIN3+ in women with ASC-US and LSIL

	Sensitivity	Specificity	PPV	NPV	AUC	Referral Rate
	%(95%CI)	%(95%CI)	%(95%CI)	%(95%CI)	(95%CI)	%(95%CI)
CIN2+						
p16/Ki67	87.5(75.3-94.1)	66.4(59.7-72.4)	37.5(29.1-46.7)	95.8(91.2-98.1)	0.769(0.701-0.838)	43.8(40.7-46.9)
HR-HPV	91.7(80.5-96.7)	55.8(49.0-62.4)	32.4(25.1-40.6)	96.7(91.7-98.7)	0.737(0.669-0.805)	53.1(50.0-56.2)
CIN3+						
p16/Ki67	89.7(73.6-96.4)	62.1(55.7-68.2)	23.2(16.4-31.8)	97.9(94.1-99.3)	0.759(0.679-0.839)	43.8(40.7-46.9)
HR-HPV	89.7(73.6-96.4)	51.5(45.1-58.0)	19.1(13.4-26.5)	97.5(92.9-99.2)	0.706(0.620-0.792)	53.1(50.0-56.2)

^a^Abbreviations: CIN2+, cervical intraepithelial neoplasia grade 2 or worse; CIN3+, cervical intraepithelial neoplasia grade 3 or worse; LBC, liquid-based cytology; CI, confidence interval; PPV, positive predictive value; NPV, negative predictive value; AUC, area under ROC curve.

Four hundred and sixty-three women were tested positive for HR-HPV in the entire population, and there were 218 CIN2+ cases in this group; the performance of HPV16/18 genotyping, LBC and p16/Ki-67 dual staining were evaluated (Table 5). In contrast with HPV16/18 genotyping, p16/Ki-67 had a higher sensitivity (92.7% *vs* 71.1, *p* < 0.0001 ) for CIN2+ and CIN3+(95.0% *vs* 79.8%, *p* < 0.0001) but a lower specificity for both endpoints(52.7% *vs* 74.7% for CIN2+, 47.7% *vs* 73.7% for CIN3+, all *p* < 0.0001). However, no significant difference was found between LBC and p16/Ki-67(all *p* > 0.05). Notably, the positivity of p16/Ki-67 was 68.7%, which means that more than 30% of women would not need referral to colposcopy if p16/Ki-67 dual staining would be used as a triage.

**Table 5:** Clinical performance characteristics of p16/Ki67 dual staining, HPV16/18 and LBC for detection of CIN2+ or CIN3+ in women who tested positive for HR-HPV

	Sensitivity	Specificity	PPV	NPV	AUC	Referral Rate
	%(95%CI)	%(95%CI)	%(95%CI)	%(95%CI)	(95%CI)	%(95%CI)
CIN2+						
p16/Ki-67	92.7(88.4-95.4)	52.7(46.4-58.8)	63.5(58.1-68.6)	89.0(82.8-93.1)	0.727(0.680-0.773)	68.7(66.5-70.9)
HPV16/18	71.1(64.8-76.7)	74.7(68.9-79.7)	71.4(65.1-77.0)	74.4(68.6-79.4)	0.729(0.682-0.776)	46.9(44.6-49.2)
LBC	94.5(90.6-96.8)	53.5(47.2-59.6)	64.4(59.0-69.4)	91.6(85.9-95.1)	0.740(0.694-0.785)	69.1(67.0-71.2)
CIN3+						
p16/Ki-67	95.0(90.7-97.3)	47.7(42.0-53.5)	53.1(47.7-58.6)	93.8(88.6-96.7)	0.713(0.667-0.759)	68.7(66.5-70.9)
HPV16/18	79.8(73.3-85.0)	73.7(68.3-78.5)	65.4(58.9-71.5)	85.4(80.4-89.2)	0.767(0.722-0.813)	46.9(44.6-49.2)
LBC	98.3(95.2-99.4)	49.1(43.4-54.9)	54.7(49.2-60.1)	97.9(94.0-99.3)	0.737(0.693-0.781)	69.1(67.0-71.2)

^a^Abbreviations: CIN2+, cervical intraepithelial neoplasia grade 2 or worse; CIN3+, cervical intraepithelial neoplasia grade 3 or worse; LBC, liquid-based cytology; CI, confidence interval; PPV, positive predictive value; NPV, negative predictive value; AUC, area under ROC curve.

## DISCUSSION

To our knowledge, this is the first study to comprehensively evaluate the performance of p16/Ki-67 dual stained cytology in primary screening and as a tool to triage women with ASCUS/LSIL or positive HR-HPV results in China. To increase the statistical power for the sensitivity estimates of p16/Ki-67, multi-center hospital-based recruitment was undertaken and a large number of women diagnosed with CIN2+ (*n* = 231, including 70 cervical cancer cases) were enrolled from the ongoing cervical cancer screening program or colposcopy clinic. To avoid the verification bias, in the screening group, three screening tests were used in parallel and women who tested positive for any of the tests were refereed to colposcopy and biopsied if needed. Four-quadrant cervical biopsies and ECC were performed to maximize ascertainment of disease if no visible lesions were found under colposcopy. The histopathology of CIN and some of cervical cancer cases were consensus expert-reviewed and p16/PR -supported. In addition, all assays in our study were run in the central laboratory and were evaluated according to the same criteria; all three screening tests were performed by using the same sample.

In the whole population, the positive rates of p16/Ki-67 increased gradually with disease severity, which correspond with results from previous studies [16, 17]. The overexpression of p16 in cervical dysplasia was reported to be associated with the transforming activity of the E7 oncoprotein of HR-HPV types through inactivation of the tumor-suppressor function of the retinoblastoma protein (pRb) [18], which may lead to malignant transformation when it occurs in DNA replication-competent cells. Therefore, simultaneous detection of p16 overexpression and expression of the proliferation marker Ki-67 within the same cervical epithelial cell should indicate HPV-related transformation. In our study, a strong association between p16/Ki-67 expression and the presence of HR-HPV types was observed, even after stratification by histology category. In particular, the p16/Ki-67 overexpression was 4-fold higher in the HPV16 and/or 18 positive group than that in the group infected with other HR-HPV types. It may due to the higher carcinogenic potential of HPV16/18 oncoproteins than that of other carcinogenic HPV types [19]. Moreover, the percent test positive of p16/Ki-67 in SCC was significantly higher than that in ADC (95.2% *vs* 71.4%, *p* < 0.01), which is believed to be less preferentially related with HPV infection. Notably, 21 cases of CIN2+ (9.1%, 21/231), including 8 cases of CIN2 (19.0%, 8/42), 8 cases of CIN3 (6.7%, 8/119) and 5 cases of cervical cancer (7.1%, 5/70) were p16/Ki-67 negative. One of the possible explanations is that not all precancer lesions progress to cervical cancer [20, 21]; however, the 5 missed HR-HPV positive cancer cases could be considered as false-negative or the failure sampling for the second slide which was used for p16/Ki-67 dual staining.

In this study, 211 CIN2+ cases were enriched to evaluate the clinical performance of p16/Ki-67 dual staining. We noted that the sensitivity of p16/Ki-67 for the detection of CIN2+ and CIN3+ in the entire population was not significantly different from that of LBC and HR-HPV test (by cobas HPV Test), but the specificity was slightly higher than that of LBC in detection of CIN2+ and HR-HPV in detection of CIN3+. It is inconsistent with previous study [13]. The possible explanation might be the different population and different Pap or HPV tests were used in these two studies. Furthermore, it is important to notify that all the Pap cytological diagnoses were made by experienced cytologists in CICAMS [22], which makes it incomparable with other studies.

All women with ASC-US and LSIL results were evaluated by biopsy in this study, allowing us to analyze the performance of p16/Ki-67 for triage of these cytology categories. For these women, p16/Ki-67 achieved sensitivity equal to HR-HPV test but with significantly improved specificity, which lead to 50% decrease to colposcopy. Our findings support that p16/Ki-67 can be a viable option for the triage of equivocal and mildly abnormal Pap cytology results, and this is consistent with previous, mostly retrospective studies or studies performed within a colposcopy clinic [16, 23-26].

Primary HPV DNA testing is believed to be efficient in cervical cancer screening, especially in China, where trained cytopathologists and health care workers are in great shortage. The challenge for HPV DNA testing as primary screening is its lower specificity. Strategies are needed to prioritize women at high cancer risk for immediate intervention. So far, several triage options were considered, including Pap cytology, HPV-genotyping, HR-HPV E6/E7 mRNA or oncoproteins test, or the use of other biomarkers to detect underlying HG CIN, such as p16/Ki-67. Previous studies have been performed to evaluate p16/Ki-67as a triage marker for HPV-positive women with normal cytology [15, 27]. Our study assessed the accuracy of p16/Ki-67 for the detection of precancer and cancer in HR-HPV positive women with complete disease ascertainment. The sensitivity of p16/Ki-67 for the detection of CIN2+ tends to be higher compared to HPV16/18 genotyping (92.7% *vs*. 71.1%) and the specificity was significantly lower (52.7% *vs*. 74.7%). Importantly, p16/Ki-67 could cut the referral rate by more than 30% compared with immediately referring of all HR-HPV positive women to colposcopy. Furthermore, there was no significant difference between the performance of p16/Ki-67 and LBC. However, in rural areas of China, where skilled cytopathologists are not available, the broad use of LBC is unrealistic. Interestingly, the recent published studies showed that the interpretation of p16/Ki-67 dual staining could be performed by staff not trained in the morphological interpretation of cytology after a short training phase, and the experimental reproducibility is quite good [28, 29], indicating the possible use of p16/Ki-67 to triage HR-HPV positive women in low or middle-income countries.

With regard to a study weakness, it is important to note that this study was conducted in a mixed population including women who attended cervical cancer screening or referred from colposcopy to enrich for CIN2+ endpoints. Hence, the results are not generalizable to the intended-use screening population. Furthermore, the current study focused on the cross-sectional assessment of the performance of p16/Ki-67 dual-stained cytology, which does not allow the evaluation of the long-term risk of HG CIN in women with negative results in the study. However, two recently published retrospective studies have shown a high long-term NPV of negative p16/Ki-67 in HPV-positive women [27, 30]. Finally, since we cannot rule out verification bias using these three tests, the sensitivity of each test may be overestimated.

In summary, in a large referral and screening combined population with excellent disease ascertainment due to a rigorous colposcopy/biopsy protocol, we found that p16/Ki-67 dual-stained cytology is promising to be used for the efficient detection of cervical precancer and cancers in various settings. Future studies are needed to investigate the management algorithm to meet local needs in terms of financial and human resources, infrastructure and capacities, societal norms and level of cancer risk reduction desired.

## MATERIALS AND METHODS

### Study population and procedures

This is a population based multicenter (*n* = 5 centers) study. It was approved by the Institutional Review Board at each participating center (Cancer Institute/Hospital, Chinese Academy of Medical Sciences(CICAMS); Shanxi Cancer Hospital; The Second Affiliated Hospital, Sichuan University; Tianjin Central Hospital of Gynecology and Obstetrics, Henan cancer hospital). The methods were carried out in accordance with the approved guidelines. From April 2014 to March 2015, women who were attending cervical cancer screening (screening group) and women who were referred for colposcopy based on one or more prior abnormal Pap test results or a positive HR-HPV test result or other clinical suspicion of cervical cancer with local biopsy-confirmed CIN2+ (enriched group) were enrolled. All participants need to meet the following criteria: aged 30 years and older, were not pregnant, had a cervix, had not been previously diagnosed with cervical cancer and were able to provide informed consent. Exclusion criteria were previous treatment for cervical diseases (including hysterectomy or destructive therapy). Written informed consent was obtained from all participants. In the screening group, cervical cytology was collected using a cytobrush and transferred to PreservCyt solution (Hologic Inc., Bedford, MA), stored at 4°C and transported to CICAMS central lab monthly for HPV DNA analysis, Liquid-based cytology (LBC) and p16/Ki-67 dual staining. Women who were positive for any screening test were referred to colposcopy and biopsy. Directed biopsy was taken from all visible cervical lesions, otherwise, the four-quadrant punch biopsy method was indicated; biopsies were taken at positions of 2, 4, 8, and 10 o'clock depending on the quadrant, and endocervical curettage (ECC) was performed. In enriched group, cervical specimens were collected before treatment. Sample collection and processing procedure were the same as for screening group.

### HPV testing

HR-HPV detection and genotyping was done on a 1ml aliquot removed from cytology specimens before LBC using the cobas HPV Test (Roche Molecular Systems Inc., Pleasanton, CA). The cobas HPV Test features automated sample preparation combined with real-time PCR technology to detect 14 HR-HPV genotypes. PCR amplification and detection occur in a single tube to detect 14 HR-HPV DNA: HPV-16 and HPV-18 individually and the other 12 types pooled (31, 33, 35, 39, 45, 51, 52, 56, 58, 59, 66 and 68). All the procedures were performed according to the recommendations of the manufacturer.

### Liquid-based cytology

Thin-layer cytology slides were prepared with ThinPrep Pap Test (Hologic Inc., Bedford, MA) and stained using the Papanicolaou method. Cytology slides were evaluated by senior cytotechnologists and results were reported according to the Bethesda 2001 classification system. Positive LBC cytology results are defined as atypical squamous cells of undetermined significance (ASC-US) or worse (LBC+), which resulted in referral to colposcopy in the cervical cancer screening group.

### p16/Ki-67 dual staining

A second cytology slide was prepared from the residual PreservCyt material for p16/Ki-67 which was conducted using the CINtec PLUS Cytology kit (Roche Tissue Diagnostics/Ventana Medical Systems, Inc., Tucson, AZ) according to the manufacturer's instructions. Samples with one or more cervical epithelial cells that simultaneously showed brownish cytoplasmic immunostaining (p16) and red nuclear immunostaining (Ki-67) were classified as positive regardless of the morphological appearance of the cells. Slides without any double-stained cells were called negative for p16/Ki-67 dual-stain cytology. All the slides were reviewed by a trained cytologist in CICAMS blindly to other tests' results.

### Histopathology

Histopathological diagnosis was made by local pathologists firstly, and then all the CIN, HPV DNA negative cervical cancer and adenocarcinoma cases were selected and reviewed by a panel of expert pathologists from each center. The final diagnosis was based on the results of the panel review. Additionally, p16^INK4A^ immunohistochemistry (IHC) staining (Roche Tissue Diagnostics/Ventana Medical Systems, Inc., Tucson, AZ) and progestogen receptor (PR) IHC staining (ZSGB-BIO, Beijing, China) were used as an adjudicator for these selected cases. For the purposes of the study, CIN2, CIN3, squamous cell carcinoma (SCC), and adenocarcinoma (ADC) were referred to as CIN2+ cases, and the other cases were referred to as CIN2- cases.

### Statistical analysis

The study targeted the recruitment of 196 CIN2+ cases, which with a clinical sensitivity of 85% for CIN2+ would result in a 95% confidence interval of approximately ±5%,i.e., 80% to 90%. Chi square of trend for proportion was calculated to test linear associations between screening methods and increasing severity of cytological and histological diagnoses. Associations between p16/Ki-67 expression and HR-HPV positive were examined using logistic regression models. Sensitivity, specificity, positive predictive value (PPV) and negative predictive value (NPV) were calculated for 2 different endpoints, CIN2+ and CIN3+. Estimates were provided with their 95% confidence intervals (95%CI). In addition, area under ROC curve (AUC) and referral rates to colposcopy based on test positivity were calculated. McNemar tests were used to compare paired matching data such as sensitivities, specificities, fractions of positive results and referral rates between different screening methods. All P values less than 0.05 (two-sided) were considered to be statistically significant. SPSS 13.0 (SPSS Inc., Chicago, IL, USA) was used for the analyses.
